# Epidemiology of neurodegenerative diseases in sub-Saharan Africa: a systematic review

**DOI:** 10.1186/1471-2458-14-653

**Published:** 2014-06-26

**Authors:** Alain Lekoubou, Justin B Echouffo-Tcheugui, Andre P Kengne

**Affiliations:** 1 Department of Neurosciences, Division of Neurology, Medical University of South Carolina, Charleston, USA; 2 Hubert Department of Global Health, Rollins School of Public Health, Emory University, Atlanta, Georgia, USA; 3 MedStar Health, Baltimore, Maryland, USA; 4 Department of Medicine, University of Cape Town, Cape Town, South Africa; 5 The George Institute for Global Health, Sydney, Australia; 6 Julius Center for Health Sciences and Primary Care, University Medical Center Utrecht, Utrecht, the Netherlands; 7 Non-Communicable Diseases Research Unit, South African Medical Research Council, PO Box 19070 Tygerberg, Cape Town 7505, South Africa

**Keywords:** Neurodegenerative diseases, Parkinsonism, Dementia, HIV-related cognitive impairment, Sub-Saharan Africa

## Abstract

**Background:**

Sub-Saharan African (SSA) countries are experiencing rapid transitions with increased life expectancy. As a result the burden of age-related conditions such as neurodegenerative diseases might be increasing. We conducted a systematic review of published studies on common neurodegenerative diseases, and HIV-related neurocognitive impairment in SSA, in order to identify research gaps and inform prevention and control solutions.

**Methods:**

We searched MEDLINE via PubMed, ‘*Banque de Données de Santé Publique’* and the database of the ‘*Institut d’Epidemiologie Neurologique et de Neurologie Tropicale*’ from inception to February 2013 for published original studies from SSA on neurodegenerative diseases and HIV-related neurocognitive impairment. Screening and data extraction were conducted by two investigators. Bibliographies and citations of eligible studies were investigated.

**Results:**

In all 144 publications reporting on dementia (n = 49 publications, mainly Alzheimer disease), Parkinsonism (PD, n = 20), HIV-related neurocognitive impairment (n = 47), Huntington disease (HD, n = 19), amyotrophic lateral sclerosis (ALS, n = 15), cerebellar degeneration (n = 4) and Lewy body dementia (n = 1). Of these studies, largely based on prevalent cases from retrospective data on urban populations, half originated from Nigeria and South Africa. The prevalence of dementia (Alzheimer disease) varied between <1% and 10.1% (0.7% and 5.6%) in population-based studies and from <1% to 47.8% in hospital-based studies. Incidence of dementia (Alzheimer disease) ranged from 8.7 to 21.8/1000/year (9.5 to 11.1), and major risk factors were advanced age and female sex. HIV-related neurocognitive impairment’s prevalence (all from hospital-based studies) ranged from <1% to 80%. Population-based prevalence of PD and ALS varied from 10 to 235/100,000, and from 5 to 15/100,000 respectively while that for Huntington disease was 3.5/100,000. Equivalent figures for hospital based studies were the following: PD (0.41 to 7.2%), ALS (0.2 to 8.0/1000), and HD (0.2/100,000 to 46.0/100,000).

**Conclusions:**

The body of literature on neurodegenerative disorders in SSA is large with regard to dementia and HIV-related neurocognitive disorders but limited for other neurodegenerative disorders. Shortcomings include few population-based studies, heterogeneous diagnostic criteria and uneven representation of countries on the continent. There are important knowledge gaps that need urgent action, in order to prepare the sub-continent for the anticipated local surge in neurodegenerative diseases.

## Background

**Figure 1:**
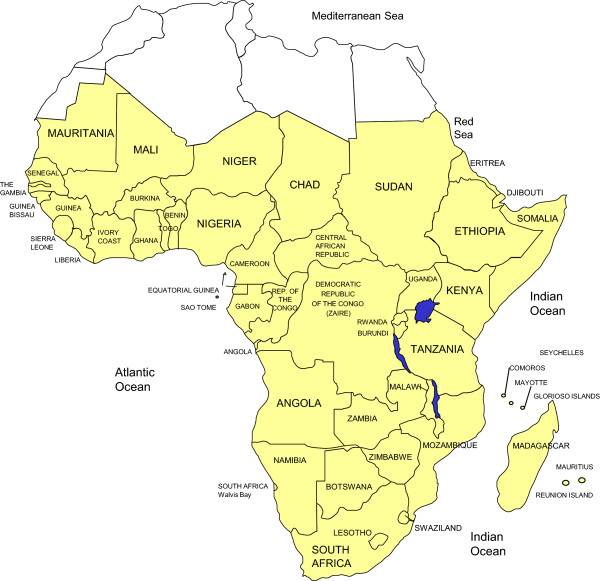
Sub-Saharan African countries.

Worldwide, populations are increasingly living longer including in developing countries, where the largest number of elderly people is currently found. In sub-Saharan Africa (SSA) (Figure 1), life expectancy at birth has increased by about 20 years between 1950 and 2010 [1]. During this same period, while the proportion of people aged 60 years and above has remained constant at around 5%, the absolute number in this group has increased by about four folds from 9.4 million in 1950 (total population 179.5 million) to 40.3 million in 2010 (total population 831.5 million). In general, population ageing has been described as a more recent phenomenon in SSA, causing figures for this region to be well below the global average [1]. However, projections suggest that the gap in life expectancy between SSA and the world average, which was around 20 years in 2010, will drop to 10 years by 2050. By this time, about 7.6% of the SSA population (estimated total 2.074 billion) will be aged 60 years and above, which in absolute number will translate into four times the 2010 estimates, and correspond approximately to 156.7 million people [2].

Population ageing is considered a global public health success, but also brings about new health challenges in the form of chronic diseases including cardiovascular diseases, cancers, as well as neurodegenerative disorders. A characterization and updated picture of the latter conditions in SSA is particularly important in view of a) the ongoing demographic transition and the resulting surge in the prevalence of neurodegenerative diseases in SSA; b) the successful roll-out of antiretroviral therapies in the region and the potential, yet unknown impact of long-term survival with HIV infection and related treatments on the occurrence of neurodegenerative disorders [3]; and c) lastly, the need for reliable data for health service planning. Recently, there have been efforts to summarize existing data for conditions like Parkinson disease (PD) [4,5] dementia [6,7] or amyotrophic lateral sclerosis [8], but not for other common neurodegenerative disorders, while there are suggestions of possible African distinctiveness in their occurrence and features [9].

We systematically reviewed the published literature on common neurodegenerative disorders and HIV-related neurocognitive impairment among sub-Saharan Africans, with the objective of describing their main features as well as clinical and public health implications.

## Methods

### Data sources

**Figure 2:**
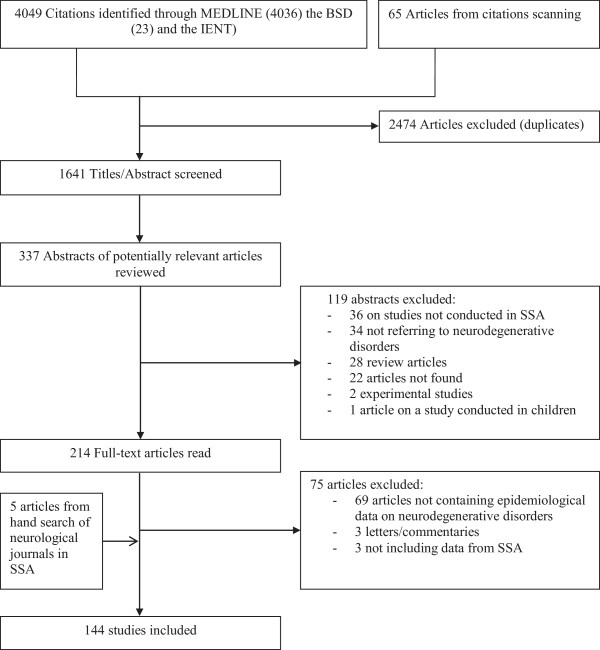
Flow of selection of studies for inclusion.

We searched MEDLINE via PubMed, and the French database ‘Banque des Données en Santé Publique’ (BDSP http://www.bdsp.ehesp.fr) for articles published until February 2013. In addition we searched the database of the ‘Institut d’Epidemiologie Neurologique et de Neurologie Tropicale’ (IENNT). We used a combination of relevant terms to search (in English for PubMed and in French for BDSP and IENNT), which are presented in Additional file 1 (except for IENNT searches for which we used ‘neuroepidemiologie’ and other themes referring to neurodegenerative diseases). Two evaluators (AL and JBE) independently identified articles and sequentially (titles, abstracts, and then full texts) screened them for inclusion (Figure 2). For articles without abstracts or without enough information in the abstract to make a decision, the full text, and where necessary supplemental materials, were reviewed before a decision was made. We supplemented the electronic searches by scanning the references lists of relevant publications, and identifying their citations through the ISI Web of Science, and by hand-searching all issues of the African Journal of Neurological Sciences. Disagreements were solved by consensus or review by a third investigator (APK).

### Study selection

We included studies conducted in a country of the SSA region (Figure 1) that reported on the following neurodegenerative diseases among adults: Alzheimer’s disease, fronto-temporal dementia, Lewy body dementia, vascular dementia, cortico-basal degeneration, multi system atrophy, Parkinson’s disease (PD), amyotrophic lateral sclerosis (ALS), Huntington disease, cerebellar degeneration, and HIV-related neurocognitive impairment. We made no restriction by study design. We excluded duplicate publications, review articles, studies conducted exclusively in pediatric populations, studies conducted exclusively on migrant populations of African descent living out of the continent. Figure 2 shows the study selection process.

We provide a rigorous appraisal of the overall data and the epidemiological studies in particular, and make recommendations regarding future approaches to measurement, notwithstanding the challenges involved in such undertakings.

### Data extraction, assessment, and synthesis

Two reviewers (AL and JBE) independently conducted the data extraction from included studies. We extracted data on study settings, design, population characteristics, measures of disease occurrence (incidence and/or prevalence), and risk factors for the various conditions examined. Given the diversity of neurodegenerative pathologies and the heterogeneity of populations assessed, we did not use a particular framework for the assessment of the quality of studies. However, whenever population-based studies and hospital-based studies had been conducted for a condition, we relied more on the conclusions of population-based studies to address relevant questions, and appropriately reported the results. We conducted a narrative synthesis of the evidence.

## Results

The study selection process is shown in Figure 2. A total of 4049 citations were identified through MEDLINE, the IENNT database and BDSP searches; 337 abstracts were evaluated in detail and 214 full-text publications reviewed. The final selection included 144 publications reporting on Parkinsonism (20 studies), dementia (49 publications), HIV-related neurocognitive impairment (47 publications), Huntington disease (19 studies), amyotrophic lateral sclerosis (15 studies), cerebellar degeneration (4 studies) and Lewy body dementia (1 study). These studies were published between 1955 and 2012, with about 50% conducted in only two countries: Nigeria and South Africa.

### Parkinson disease, other Lewy body diseases and fronto-temporal dementia

Twenty studies reported on Parkinsonism (Table 1), including five community-based and sixteen hospital-based. Four were case–control in design and all the others were cross-sectional studies, including reviews of medical records. These studies were conducted in seven countries including Nigeria (ten studies), South Africa (four studies), Tanzania (two studies), Ethiopia, Ghana, Cameroon and Zimbabwe (one studies each). The number of participants with PD ranged from two to 32 and the prevalence from ten to 235/100,000 in community-based studies. The number of participants with Parkinsonism ranged from four to 397, and the prevalence of Parkinsonism varied from 0.41 to 7.2% of neurological admissions/consultations in hospital-based studies. The proportion of men among those with PD ranged from 53 to 100%, and age ranged from 30 to >100 years. Age at the clinical onset of the disease ranged from 17 to 90 years. The clinical types of the disease were largely dominated by Parkinson disease (38 to 100%).

**Table 1:** Overview of studies on Parkinsonism and risk factors in sub-Saharan African countries

**Author, year of publication**	**Country**	**Setting**	**Design/period of study**	**Population characteristics**	**Diagnosis criteria**	**Prevalence**	**Profile of parkinsonism patients**	**Comments**
Bower [10], 2005	Ethiopia	Hospital	Cross-sectional 2003-2004	720 patients; 109 (15 · 1%) with movement disorders including 71 men; age 52 y. (13–80)	Not provided	72/1,000 of all admissions (PD: 64/1,000)	N:52; PD:88%	Review of medical files/outpatient neurology clinic.
							Age (at onset): 57y (30–80)	
							Men: 75%	
Akinyemi [11], 2008	Nigeria	Hospital	Case–control 2005-2005	51 patients (men 37) with PD and 50 controls	UKPDS Brain Bank criteria	NA	N:51; PD: 100%	22% patients with PD had cognitive dysfunction, with age at PD onset as sole predictor of cognitive dysfunction.
							Age (at onset): 70y (41–80)	
							Men:72%	
Cosnett [12], 1988	South Africa	Hospital	Cross-sectional 1979-1985	2638 patients	Clinical (Bradykinesia, rigidity, resting tremor and postural instability)	5.3/1,000	N:14; PD: 100%	Retrospective review of medical files/outpatient clinic
							Age: NA	Blacks: 1.5/1000
							Men: NA	Indians: 12.6/1000
								Whites: 23.1/1000
Dotchin [13], 2008	Tanzania	Community	Cross-sectional	161,071 inhabitants	UKPDS Brain Bank criteria	Overall: 40/100,000	N: 32; PD:100%	Prevalence is adjusted to UK population. Mean duration 5.1 y
						Men: 64/100,000		
						women: 20/100,000	Age (at onset): 69y (29–90)	
							Men: 72%	
Schoenberg [14], 1988	Nigeria	Community	Cross-sectional	Black population aged 40 + 3412 participants	Clinical	Age adjusted: 67/100,000	N: 2; PD:100%	
							Age: NA	
							Men: NA	
	USA	Community	Cross-sectional	Black population aged 40 + 3521 black participants and 5404 white participants.	Clinical	Age adjusted:	N: 12; PD: 100%	
							Age: NA	
						Blacks: 341/100,000	Men: NA	
						Whites: 352/100,000		
Winkler [15], 2010	Tanzania	Hospital	Cross-sectional	n = 8676 patients admitted (740 with neurological diseases)	UKPDS Brain Bank criteria	1/1,000 (all patients)	N: 8; PD:37%	
			2003			11/1,000 (Patients with neurological diseases		
							Age: ≥32 y	
							Men: 100%	
		Community	Cross-sectional	1569 people, age 50–110 years	UKPDS Brain Bank criteria	235/100,000	N: 0	None of the 18 screened-positive was confirmed as having PD. Poisson distribution used to estimate the prevalence.
			2003-2005					
Kengne [16], 2006	Cameroon	Hospital	Cross-sectional	4041 patients in a neurology clinic145 (3.9%) had neurodegenerative diseases	Not provided	488/1,000 of all neurodegenerative diseases; 10.1/1,000 of all neurologic consultation	N: 41; PD 100%	4 selected neurodegenerative brain disorders: dementia, PD, ALS, chorea
			1993-2001				Age: 15-84 y	
							Men: 73.2%	
Lombard [17],1978	Zimbabwe	Hospital	Cross-sectional	Total patients admitted: 83,453 blacks, 34,952 whites	Not provided	Blacks: 0.21/1,000	N: 50 (17 blacks)	Retrospective review of medical files
						Whites: 2.83/1,000	Age/men: NA	
Osuntokun [18], 1979	Nigeria	Hospital	Cross-sectional	217 patients with parkinsonism	Not provided	NA	N: 217; PD 38%	All patients evaluated by the authors
			1966-1976				Age: median 51-70 y,	
							Men:75%	
Osuntokun [19], 1987	Nigeria	Community	Cross-sectional	Total participants surveyed: 18,954	Not provided	10/100,000	N. 2; PD 100%	Screening Questionnaire developed by author
			1985				Age/men: NA	
Haylett [20], 2012	South Africa	Hospital	Cross-sectional	229 patients with PD including 163 whites (71%), 45 mixed ancestry (20%), 17 blacks (7%) and 4 Indians (2%)	UKPDS Brain Bank criteria	NA	N: 229; PD 100%	Mutation in the Parkin gene
							Age (at onset): 54 y (17–80)	Homozygous or compound heterozygous mutations: 7 patients
								Heterozygous variant: 7
							Men: % NA	
Ekenze [21], 2010	Nigeria	Hospital	Cross-sectional	8440 admission in the medical ward; 1249 had neurological diseases (men 640)	Not specified	21.9/1000 of al neurological admissions	N: 14	
			2003-2007				Age ≥ 70 y (71%)	
							Men: 28.6%	
Owolabi [22], 2010	Nigeria	Hospital	Cross-sectional	6282 admission in the medical ward; 980 had neurological diseases (men 586)	Clinical: any 3 out of tremor, rigidity, Akinesia/bradikinesia/postural and instability	4.1/1,000 of all neurological admissions	N: 4	
			2005-2007				Age: (50–68)	
							Men; 100%	
Okubadejo [23], 2004	Nigeria	Hospital	Case–control	33 participants (men 25, mean age 60 y) with PD and 33 match controls	Any 3 out of tremor, rigidity, Akinesia/bradikinesia/postural and instability	NA	N: 33	Case fatality rate was higher in PD (25% vs. 7.1%), Factors associated with increased mortality: advanced age and disease severity
							Age (at onset): 36-80y	
							Men: 75%	
Okubadejo [24], 2005	Nigeria	Hospital	Case–control	28 participants (men 21, mean age 63 y) with PD and 28 match controls	Any 2 out of tremor, rigidity, Akinesia/bradikinesia/postural and instability, exclusion of other causes of parkinsonism	NA	N: 28; PD 100%	Autonomic dysfunction rate was higher in PD (61% vs. 6%),
							Age (at onset): 37-76 y	
							Men: 76%	
Okubadejo [25], 2010	Nigeria	Hospital	Cross-sectional	124 participants with Parkinsonism in a neurology clinic	Any 3 of the following: tremors, rigidity, bradykinesia, and postural or gait abnormality	15/1,000 of all neurological consultations	N: 98; PD 79%	Other causes of parkinsonism n(%): Vascular/drug induced/MSA/LBD: 9(35)/5(19)/4(15)/3(11)
			1996-2006					
							Age (at onset): 61y Men: 76.5%	
Keyser [26], 2010	South Africa	Hospital	Cross-sectional	154 patients with PD including 51 whites (35%), 45 Afrikaners (31%), 29 mixed ancestry (20%), 17 blacks (12%) and 3 Indians (2%).	UK Parkinson’s Disease UKPDS Brain Bank criteria	NA	N: 154; PD 100%	16 sequence variants of the PINK1gene identified: 1 homozygous mutation (Y258X), 2 heterozygous missense variants (P305A and E476K), and 13 polymorphisms
							Age (at onset): 52 y	
							Men: 62%	
Van Der Merwe [27], 2012	South Africa	Hospital	Cross-sectional	111 patients with early onset PD (men 71) and 286 with late onset PD (men 62%) from a movement disorder clinic	UKPDS Brain Bank criteria	NA	N: 397; PD 100%	A positive family history was associated with a younger age at onset.
			2007-2011				Age (at onset): 57 y Men: 248	
Femi [28], 2012	Nigeria	Hospital	Cross-sectional	1153 participants in 2 Neurologic clinics; 96 (men: 74) had parkinsonism	presence of at least three of the four cardinal features of tremors, rigidity, bradykinesia, and postural or gait abnormality	69.4/1,000 of all neurological consultations	N: 96; PD (83.3%)	
			2007-2011					
							Age: 58 y	
							Men: 63.5%	
Cilia [29], 2012	Ghana	Hospital	Case–control	54 participants with PD and 46 healthy participants	UKPDS Brain Bank criteria	NA	N: 54; PD 100%	Leucine-rich repeat kinase 2 (LRRK2) gene found in no participants
							Age (at onset): 59 y (30–83)	
							Men: 61%	

NA: Not available; PD: Parkinson’s disease; UK: United Kingdom; USA: United States of America; y: years.

The most commonly used tool to diagnose PD was the UKPDS Brain bank criteria and population-based (hospital-based) prevalence for the studies that applied those criteria ranged from 40 to 235/100,000 (11 to 69.4/1,000 neurological consultations). In general risk factors were not investigated across studies, although one study found that 38% of patients with Parkinsonism had atherosclerosis and 8% had encephalitis [18].

We found three cases of Lewy body dementia in a retrospective study in Nigeria, and one case in a retrospective study in Senegal representing respectively 1.2/100,000 of admission over a period of 10 years [30] and 7.5/1000 of participants in a specialized memory clinic [31].

The prevalence of fronto-temporal dementia has been reported in two hospital-based studies conducted in Neuropsychiatric clinics in Nigeria (prevalence rate: 1.7/100,000 of all admissions) and in Senegal (prevalence rate: 7.5/1000 of all participants evaluated for memory impairment) [30,31].

### Dementia

(Table 2) summarizes the 49 publications that reported on dementia. These include 18 hospital-based, 30 community-based publications and one publication from a nursing home. Two were case–control in design, seven were cohort-studies and 40 were cross-sectional, including two autopsy studies. These publications reported on studies conducted in eleven countries: Nigeria (33 publications), Senegal (four publications), Kenya and Tanzania (three publications each), Benin, Central African Republic, Congo republic, (two publications each), South Africa, Cameroon and Zambia (one publication each). In addition, there were seven publications on multicenter studies including African American participants in the USA and participants from African countries [32-37]. The overall study size varied from 56 to 2494 in community-based studies and from 23 to 240,294 in hospital-based investigations. The prevalence of dementia ranged from <1% to 10.1% in population-based studies [32,34-57] and from <1% to 47.8% in hospital-based studies [16,21,30,33,38,58-69].

**Table 2:** Overview of studies on dementia and risk factors in sub-Saharan Africa

**Author, year of publication**	**Country/setting**	**Design/period of study**	**Population characteristics**	**Diagnostic criteria**	**Incidence**	**Prevalence (%)**	**Risk factors**
Lambo [58], 1966	Nigeria	Retrospective/Cross-sectional, 1954-1963	328 participants (26% ≥60 y.)	Not provided	NA	Senile dementia*:	NA
	Hospital						
						Overall: 26%, Men: 18.9% Women: 30.5%	
			75 cases of dementia (21 men)				
Ben-Arie [39], 1983	South Africa	Cross-sectional, 1982	139 participants aged ≥65 y.	MMSE/ICD-8 codes	NA	Any (severe) dementia 8.6% (3.6%)	NA
	Community						
Makanjuola [59], 1985	Nigeria	Cross-sectional 1979-1982	51 (5.2% of new consultations); age ≥60 y.	ICD-9 codes	NA	Dementia 11.2%	NA
	Hospital						
Gureje [60], 1989	Nigeria	Cross-sectional, 1984	1914 patients;	ICD- 9 codes	NA	No case of dementia	NA
	Community						
Ogunniyi [40], 1992	Nigeria	Cross-sectional	930 participants; age ≥40 y. (293 aged ≥65 y.); No case of dementia	DSM-III-R criteria	NA	No case of dementia	NA
	Community						
Osuntokun [61], 1994	Nigeria, hospital Autopsy study	Cross-sectional 1986- 1987	111 brains autopsied including 85 patients aged ≤60 y.	Beta A4 amyloid on brain tissues	NA	Heavy/moderate/mild plaque load: 0/6.3/18.9%	NA
Osuntokun [41], 1995	Nigeria, community	Cross-sectional	56 subjects (17 with dementia and 12 with AD); age ≥65 y.	Dementia –CSID	NA	APOE ϵ4 allele in dementia/AD/controls 17.6/16.7/20.5%.	NA
				AD - NINCDS-ADRDA criteria			
Osuntokun [38], 1995	Nigeria, hospital Autopsy study	Cross-sectional	198 brains were autopsied	senile plaque, neurofibrillary tangle, and amyloid vascular degeneration	NA	No evidence of NFT or senile plaque	NA
		1986- 1987	Including 45 (23%) ≥65 year				
Hendrie [32], 1995	Nigeria, community	Cross-sectional	2494 participants, age ≥65 y., Dementia -28, AD - 18, VaD - 8.	Dementia: CSID/DSM-III-R/ICD-10/AD: NINCDS-ADRDA criteria	NA	Dementia - Overall/65-74/75-84/≥85 y:	
		1992-1993				2.3/0 · 9/2.7/9.6;	
						AD - 1.4/0.5/1.7/5.9%	
	Indianapolis-USA, community & nursing home	Cross-sectional	2212participants, aged ≥65 y. (community) and 106 (nursing home)	Dementia: CSID/DSM-III-R/ICD-10/AD: NINCDS-ADRDA criteria	NA	Dementia Overall/65-74/75-84/≥85 y:	NA
		1992 - 1993					
						8.2/2 · 6/11.4/32.4%	
						AD -6.2/1.6/8.0/28.8%	
Ogeng'o [33], 1996	Tanzania, hospital	Cross-sectional	12 Non-demented subjects aged 45–83 y.	senile plaque, neurofibrillary tangle, and cerebral amyloid angiopathy	NA	Amyloid β plaques:17%	NA
1996		Autopsy study					
						Neurofibrillary Tangles: 17%; Cerebral Amyloid angiopathy: 17%	
	Kenya, hospital	Cross-sectional Autopsy study	20 Non-demented subjects aged 45–70 y.	Senile plaque, neurofibrillary tangle, and cerebral amyloid angiopathy	NA	Amyloid β plaques: 15%; Neurofibrillary Tangles: 15%; Cerebral Amyloid angiopathy: 15%	NA
	USA-Cleveland, Hospital	Cross-sectional/Autopsy study	20 Non-demented subjects aged 48–84 y.	Senile plaque, neurofibrillary tangle, and cerebral amyloid angiopathy	NA	Amyloid β plaques: 20%; Neurofibrillary: 15%; Cerebral Amyloid angiopathy: 20%	NA
Ogunniyi [42], 1997	Nigeria, community	Cross-sectional	2494 participants aged >65 y screened, 28 with dementia.	Screening: CSI-D)	NA	Any/ AD/ vascular dementia - 1.1/0.7/0.3%	N A
		1992-1994					
				Dementia: DSM-III-R and ICD-10 codes			
				AD: NINCDS-ADRDA			
Sayi [62], 1997	Tanzania, hospital	Cross-sectional	24 demented and 286 non-demented participants aged 50–89 y.	Swahili modified MMSE	NA	Prevalence of ϵ4 allele of APOE: Demented - 25%; non demented - 21%	NA
	Kenya, hospital	Cross-sectional	22 demented and 60 non-demented participants aged ≥65 y.	Swahili modified MMSE	NA	Prevalence of ϵ4 allele of APOE: Demented - 42%, non-demented - 27%	NA
Baiyewu [63], 1997	Nigeria, Nursing home	Cross-sectional	23 participants (in a nursing home) aged 66–102 y.; 11 women	DSM-III-R/AGECAT	NA	Any dementia (AD) - 47 · 8% (26 · 1%)	NA
		1994					
Hall [34],1998	Nigeria, community	Case–control	2494 participants; age ≥ 65 y.;	Screening: CSID	NA	18 cases of possible or probable AD1.4%	age (OR = 1.15; 95%
			423 clinically assessed after screening,				
							CI = 1.12-1.18) and female gender (OR = 13.9; 95% CI = 3.85-50.82)
				Dementia: DSM-III-R/ICD-10/AD: NINCDS-ADRDA			
	USA–Indianapolis, community	Case–control	2212 participants; age ≥ 65 y.;	Screening: CSID	NA	Possible/probable AD 6.2%	age, family history of dementia, education; rural residence
				Dementia: DSM-III-R/ICD-10/AD: NINCDS-ADRDA			
			351 clinically assessed after screening,; 49 (men 17) diagnosed with AD				
Uwakwe [70], 2000	Nigeria, Hospital	Cross-sectional	119 participants; age ≥65 y; 3 had dementia	Geriatric Mental State and/ICD-10	NA	2.8%	NA
		1995-1996					
Ogunniyi [43], 2000	Nigeria, community	Cross-sectional 1992-1994	2494 participants, age ≥65 y.; 28 with dementia (men: 8) including 18 with AD, 8 with vascular dementia	Screening: CSID	NA	Any dementia 2.3%	Age (OR: 1.15), female gender (13.9), living with others (OR: 0 · 06)
				Dementia: DSM-III-R/ICD-10			
				AD: NINCDS-ADRDA		AD: 1.4%	
						E4 allele in AD (normal subjects) 34.2% (21.8%)	
	Indianapolis-USA, community	Cross-sectional	2212 participants, age ≥65 year; 65 with dementia including 49 with AD, 10 with vascular dementia	Screening: CSID	NA	Dementia (AD) overall/65-74/75-84/≥85 y – 8.2 (6.2)/2.62 (1.58)/ 11.4 (8.0)/32 · 4% (28.8%);	Age, rural residence, family history of dementia, education
				Dementia: DSM-III-R/ICD-10			
		1992-1994					
				AD: NINCDS-ADRDA			
Hendrie [35], 2001	Nigeria, community	Prospective cohort Baseline survey in 1992-1993	2459 participants included after the first visit; 1303 (men 461) completed the follow-up; age ≥65 y.	Screening: CSID	Dementia: 13.5/1,000	NA	NA
				Dementia: DSM-III-R/ICD-10			
				AD: NINCDS-ADRDA	AD**:** 11.5/1000		
	USA-Indianapolis, community	Prospective cohort	2147 African-Americans included after the first visit; 1321 (men 417) completed the follow-up; age ≥65 y.	Screening: CSID	Dementia (AD)	NA	NA
		Baseline survey in 1992-1993					
				Dementia: DSM-III-R/ICD-10/AD: NINCDS-ADRDA			
					32.4/1,000 (25.2/1,000)		
Baiyewu [44], 2002	Nigeria, community	Prospective cohort baseline survey in 1992-1993	2487 participants; age ≥65 y.;	Screening: CSID	Conversion from CIND to dementia 16 · 1%; From CIND to normal 25 · 3%	NA	Sex
				Dementia: DSM-III-R/ICD-10			
			423 clinically assessed after screening; 152 diagnosed with CIND; 28 (men 7) with dementia, 87 followed up for 2 years.				
Perkins [36], 2002	Ibadan-Nigeria community	Prospective, 1992-1993	2487 participants; age ≥65 y;	Screening: CSID	NA	1.8%	Dementia associated with mortality
				Dementia: DSM-III-R/ICD-10			
			423clinically assessed after screening				
	Indianapolis-USA, Community	Prospective Baseline survey in 1992-1993	2212 participants; aged ≥65 y.;	Screening: CSID		4.9%	Dementia associated mortality (adjusted RR: 2 · 05)
			342 clinically assessed after screening				
				Dementia: DSM-III-R/ICD-10			
Lane [37], 2003	Nigeria Community	Prospective 8.7 y follow up Baseline 1992-1993	968 participants (271 aged ≥75 y.);	Screening: CSID	NA	NA	ApoE*ϵ*4 alleles not associated with increased mortality
			23with dementia at follow-up				
				Dementia: DSM-III-R/ICD-10			
	Indianapolis-USA, Community	Prospective 9.5 y. Baseline 1992-1993	353 participants (17 4 aged ≥75 y.); 17 with dementia at follow-up	Screening: CSID	NA	NA	ApoE*ϵ*4 associated with increased mortality for patient under 75 year
				Dementia: DSM-III-R/ICD-10			
Ogunniyi [45], 2005	Nigeria, Community	Cross-sectional/1992- 1998	98 demented subjects; age ≥65 y.	Screening: CSID	NA	AD: 82% of all cases	NA
				Dementia: DSM-III-R/ICD-10			
						VaD: 11.1% of all cases	
Kengne [16], 2006	Cameroon,	Cross sectional,	4041 neurologic consultations	Not provided	NA	0.4% (all neurologic admission), 19% (neurodegenerative diseases)	NA
	Hospital	1993-2001					
			145 with neurodegenerative diseases				
			16 (men 14) with dementia, mean age 67.8 y.				
Gureje [46], 2006	Nigeria, Community	Cross-sectional, 2003-2004	2152 participants at baseline with a respondent rate of 74% (1904 participants). Aged 65 year or older.	adapted 10-Word Delay Recall Test (10-WDRT)10	NA	Overall: 10.1%;	Female gender, Increasing age, alcohol
						Female: 14.6%	
						Men: 7.0%	
Gureje [71], 2006	Nigeria Community	Cross-sectional,	2245 DNA samples, 830 had a diagnosis	Screening: CSID	NA	Any dementia (16.9%	E4 allele in AD (normal subjects) 26 · 0% (21 · 7%)
				Dementia: DSM-III-R/ICD-10			
						AD: 14.8%	
Ogunniyi [72], 2006	Nigeria, Community	Case–control	62 participants with AD (Men 16.1%, mean age 82 y) and 461 non demented (men 33.2%, mean age 77 y)	Screening: CSID	NA		Age (OR 1 · 07)
				Dementia: DSM-III-R/ICD-10/AD: NINCDS-ADRDA			
							Rural to age (OR 2 · 93) Hypertension (OR 0 · 33)
	Indianapolis-USA, Community	Case–control	89 participants with AD (men 30.3%, mean age 83 y), mean age 77 y) and 381 non demented (Men 31.2%, mean age 78 y)	Screening: CSID	NA		Age (OR 1.09
							Rural to age (OR 2.08)
				Dementia: DSM-III-R/ICD-10/AD: NINCDS-ADRDA			Alcohol consumption (OR 0.49)
Uwakwe [64], 2006	Nigeria, Community	Cross-sectional	30 patients (men 12) with dementia and their caregivers (total 30)	Not provided	NA	N:52;	
		2003-2005					
						AD: not provided	
						Men: 12	
Ochayi [47], 2006	Nigeria, Community	Cross-sectional 2002	280 participants; age ≥65 y.;	CSID	NA	Overall dementia: 6.4%	Female gender,
							Lower body mass index, age, NSAIDS
						65-74 year old: 5.2%	
			18 (men 2) with dementia			≥85 year 16%.	
Hall [48], 2006	Nigeria, Community	Cross-sectional	1075 participants; age ≥ 70 y. 29 (men 5) with AD,	NINCDS-ADRDA	NA	NA	Total- or LDL- cholesterol in individuals without the *APOE*-ϵ4 allele
Uwakwe [73], 2009	Nigeria, community	Cross-sectional	914 (men 432) participants, age ≥65 y; 87 with ≥2 tests memory tests impaired	Memory impairment assessed by MMS, CISD and 10 word list immediate and delayed recall	NA	9.9%	NA
Guerchet [50], 2009	Benin Community	Cross-sectional	502 (men 156) participants, aged ≥65 y; 52 with cognitive impairment	Screening: CSI-D	NA	Cognitive impairment	Age, current depressive disorder, absence of the APOE ϵ 2
						Overall: 10.4%; men 7.7 women 11.5%	
				Dementia: DSM-IV			
				AD: NINCDS-ADRDA		Dementia Overall: 2.5%, men 0.6% women 3.4%	
			13 (men 1) with dementia				
Toure [67], 2009	Senegal	Cross-sectional	872 participants; age ≥55 y.	DSM-IV-R	NA	Overall 6.6%	Age, social isolation, history of stroke, epilepsy, family history of dementia, Parkinson’s disease
	Hospital	2004-2005	58 cases of dementia				
Napon [68], 2009	Burkina Fasso	Cross-sectional	15815 (2396) out (in) participants; age ≥15 y.; 72 (and 53 inpatients) with dementia; AD: 7; VaD: 19 cases	DSM-IV	NA	outpatients: 0.45% inpatients: 0.22%	NA
	Hospital						
Guerchet [49], 2010	Central African Republic Community	Cross-sectional	509 interviewed; 496 (men 218) included in final sample, age ≥65 y.	Screening: CSID	NA	Overall: 8.1%, men 2.7%, women 12.2%	NA
		2008-2009					
				Dementia: DSM-IV			
			188 with cognitive impairment and 40 (men 6) with dementia (mean age 76 y.); 33 (men 3) with AD and 7 (men 2) with VaD	AD: NINCDS-ADRDA Hachinski scale,			
	Republic of Congo Community	Cross-sectional	546 interviewed; 520 (men 198) included in final sample, age ≥65 y. 148 with cognitive impairment and 35 (men 9) with dementia (mean age 79 y.); 24 (men 7) with AD and 11 (men 3) with VaD	CSID/ DSM-IV and NINCDS-ADRDA Hachinski scale		Overall: 6.7%, men 4.5%, women 8.1%	NA
		2008-2009					
Chen [65], 2010	Kenya	Cross-sectional	100 participants; age ≥ 65 y.	CSI-D using a version in Kikuyu.	NA	Apo ϵ4 allele frequency:	NA
	Hospital						
						Demented 31.3%, non-demented 32.2%	
			84 controls (men 38) and 16 with dementia participants (men 7)				
Ekenze [21], 2010	Nigeria	Cross-sectional	8440 admissions; 1249 (men 640) with neurological diseases (age range18-83 y.); 38 (men 23) with dementia	Not specified	NA	3%	NA
	Hospital	2003-2007					
Siddiqi [69], 2009	Zambia	Cross-sectional	443 inpatients (men 219); median age 39 y., 67 with HIV; 368 outpatients (men 168); median age 39 y., 58 with HIV; 36 with dementia	Not specified	NA	Dementia:	Dementia in HIV + patients 8 (13.8%) vs. general population 9 (2.9%) (p = 0.002)
						Overall: 4.4%	
	Hospital	2006					
Yusuf [74], 2011	Nigeria Community	Cross-sectional	322 participants (men 128); mean age: 75.5 y	Screening: CSID/CERAD/SDT	NA	Dementia: 2.8%	Age
						AD: 1.9%	
						VaD: 0.6%	
				Dementia: DSM-IV and ICD-10			
			9 cases of dementia (men 3); mean age: 82.4 y	LBD: McKhan clinical criteria			
				FTD: McKeith clinical criteria			
Gureje [51], 2011	Nigeria, Community	Prospective Cohort Baseline 2003-2004	2,149 participants at baseline	10-Word Delayed Recall	21.80/1,000	NA	Poor social engagement, rural residence, low economic status, female gender, age.
				Test (cut off of 18)			
			1,408 at 39 months follow-up; 85 (among ≥65 y.) developed dementia				
Ogunniyi [52], 2011	Nigeria Community	Cohort study	1559 participants aged > 65 year without dementia a baseline. 136 (men 33) with dementia (mean age 83.1 y.) at follow-up; 255 with MCI	Dementia: DSM-III-R and ICD-10	Dementia: 8.72/1,000/year	NA	Low BMI
		1992-2007			MCI: 16.35/1000/year		
Ogunniy [53], 2011	Nigeria Community	prospective cohort baseline 1992	2718 participants interviewed	Dementia: DSM-III-R and ICD-10	Dementia/AD/VaD (per 1,000/year) 11.50/9.50/1.10	NA	Higher SBP, DBP and PP
			1753 (age ≥65 y.) in the final sample				
			120 (men 30) with dementia (mean age 83.8 y.); 99 with AD; 11 with VaD				
Paraïso [56],	Benin Community	Cross-sectional	1,139 (men 523) participants; age ≥65 y.; 42 (men 13) with dementia (mean age 79 · 1 y)	Screening: CSI-D	NA	Dementia Overall 3.7% men 1.1% women: 2.5%	NA
2011							
		2008					
				Dementia: DSM-IV			
			32 with AD, 105 with CIND				
				AD: NINCDS-ADRDA		AD Overall 2.8%	
						VD Overall 0.8%	
				VaD: NINCDS-AIREN			
Amoo [30],	Nigeria	Cross-sectional	240,294 participants	Dementia: ICD-10	NA	Dementia: 45/100,000	NA
2011							
						AD; 25 · 8/100,000	
	Hospital	1998-2007				VaD: 7 · 4/100,000	
			108 (men 51) with dementia (mean age: 70.1); 62 (men 24) with AD; 18 (men 13) with VaD; 4 (men 2) with mixed forms;	ADNINCDS – ADRDA			
				VaD: NINCDS –AIRENS			
				LBD: McKeith criteria,			
				FTD: Lund and Manchester Criteria			
			4 (men 2) with FTD; 3 (men 0) with DLB; 13 (men 2) with unclassified dementia				
Ndiaye [31], 2011	Senegal	Cross-sectional	132 patients seen at a memory clinic (men 41, mean age: 67 y	Screening: “Test du Senegal”/modified HodKinson test	NA	MCI: 14.4%	NA
	Hospital						
		2004-2005					
			57 with dementia; 37 with AD, 10 with VaD, 5 with FTD and 1 with LBD.			Dementia: 43.2%	
						AD: 64.7% of all cases of dementia	
				MCI: Petersen criteria			
				Dementia: DSM-IV			
Coume [75], 2012	Senegal	Cross-sectional	872 (men 546) participants aged >55 y; mean age 67 · 2 y	Test du Senegal	NA	Cognitive impairment 10.8%	NA
	Hospital	2004-2005	94 (men 65) with cognitive impairment (74 aged > =65 y)				
Baiyewu [54]., 2012	Nigeria	Cross-sectional/2001 and 2004	21 (men 4) participants with normal cognition (mean age 82.8 y.)	Screening: CSID	NA	NA	NA
				Dementia: DSM-III-R/ICD-10			
	Community		53 (men 4) with cognitive impairment (mean age 80.9); 34 (men 6) with dementia (mean age 83.3 y)	AD: NINCDS-ADRDA			
Toure [66], 2012	Senegal	Cross-sectional	507 participants; age ≥65 y.	Screening: Aging in Senegal Questionnaire	NA	8.9%	advanced age (Age ≥80 y, OR 4.3, 95% CI 1.4-13), illiteracy, epilepsy, family history of dementia
			45 with dementia	DSM-IV-R			
	Hospital	2004-2005					
Longdon [57], 2012	Tanzania	Cross-sectional	1198 (men 525) participants; age ≥70 y; 78 with dementia	Screening: CSI-D	NA	6.4%	Advanced age
	Community	2010					
				DSM-IV-R			
Onwuekwe [76], 2012	Nigeria	Cross-sectional	135 participants (men: 79), aged between 16–76 y	MMSE (cut off of 17 for MCI)	NA	MCI: 5.9%	
	Hospital	2004					
			8 with MCI				
Guerchet [55], 2012	Central African Republic, Congo	Cross-sectional	509 interviewed; 496 (men 218) included in final sample; age ≥65 y.; 188 with cognitive impairment	Dementia: DSM-IV-R/AD: NINCDS-ADRDA	NA	Dementia: 7.4%	Hypertension, low BMI, depressive symptoms, change of residence, age (OR 2.59, 95% CI, early death of one parent, female gender
		2008-2009					
	Community					AD: 5.6%	
			546 interviewed; 520 (men 198) included in final sample; age ≥65 y.; 148 with cognitive impairment				
			Overall 75 (men 15) had dementia 18 with vascular dementia				

AD: Alzheimer’s disease; APOE: Apolipoprotein E; ICD: International Classification of Disease; BMI: Body Mass Index; CI: confidence Interval; CIND: Cognitive Impairment and No Dementia; CSID: Community Screening Interview for Dementia; DSM-III-R: Diagnostic and Statistical Manual 3^rd^ edition revised; MMSE: Mini Mental State Examination; NA: Not available; NFT: Neurofibrillary tangle; NINCDS/ADRDA: National Institute of Neurological and Communicative Disorders and Stroke and the Alzheimer's Disease and Related Disorders Association; OR: Odd ratio; SCEB: short cognitive evaluation battery; USA: United States of America; VaD: Vascular dementia; y: years.

The proportion of men among those with dementia was 7.1 to 69.1%. The mean age of participants ranged from 70.1 to 83.8 years. When provided, age at clinical diagnosis of disease ranged from 80.7 to 83.8 years. Alzheimer disease was the most common form of the disease, representing 57.4 to 89.4 % of all cases [30-32,34,42,45,55,56,63,71,74], followed by vascular dementia 5.7 to 31.0% of cases [30,31,45,56,74]. Four publications in Nigeria provided incidence data for dementia ranging from 8.7 to 21.8 cases per 1000 per year [35,51-53]. Incidence of Alzheimer disease ranged from 9.5 to 11.5 per 1000 per year [35,53].

The most commonly used tool for dementia screening was the Community Screening Interview for Dementia (CSID) questionnaire applied in 20 publications [32,34,36,37,41-43,45-47,49,50,54,56,65,70]. The diagnosis of dementia mainly relied on the DSM-III-R/DSM-IV and ICD-10 classification [30,32,34-37,40,42-46,52-54,63,70]. The diagnosis of Alzheimer’s disease was based on the National Institute of Neurological and Communicative Disorders and Stroke and the Alzheimer’s Disease and Related Disorders Association (NINCDS/ADRDA) criteria [30,32,34,35,41,43,48,50,52-56,75]. Population-based studies that used DSM-III/DSM-IV and ICD-10 for dementia reported prevalences ranging from 1.1 to 8.1% [32,35,42,49,55-57,65,67,74] (ref 13, 16, 23, 30, 36–38, 48, 50, 118). Likewise the prevalence of Alzheimer’s disease ranged from 0.7 to 5.6% based on NINCDS/ADRDA criteria [35,42,55].

Risk factors for dementia were reported in 14 publications. The following were associated with an increased risk of dementia: age (twelve publications), female sex (five publications), low body mass index (three publications), anxiety/depression (three publications), hypertension (three publications), social isolation (two publications), lifetime history of alcohol consumption, elevated total- or LDL cholesterol in those without Apo E ϵ4 (one publication), low socio-economic status, history of stroke and family history of dementia (one publication). The following characteristics were inversely associated with dementia: living with others, use of non-steroidal anti-inflammatory drugs and absence of Apo E ϵ2. Some risk factors were more strongly related to the disease. These include age, which increased the risk of dementia by five to 16% across groups [34,43], but this effect was much higher after the age of 60 years, more than 100% increase risk especially after the age of 75 [46,50,51,55,66,67]. Female sex, low level of education (<6 years), rural residence and family history increased the risk of dementia by >100% [34,43,46,55,56,66].

### HIV-related neurocognitive impairment

Fifty-one hospital-based studies (47 publications) reported on HIV-related neurocognitive impairment (Table 3), of which ten were case–control, six cohort and 31 cross-sectional. These studies were conducted in 14 countries including South Africa (14 studies), Uganda (eight studies), Nigeria (six studies), Zambia and Kenya (four studies each), Cameroon and Democratic republic of Congo (three studies each) Ethiopia and Malawi (two studies each), Central African Republic, Botswana, Guinea Bissau, Tanzania and Zimbabwe (one study each). A total of 33 out of the 47 selected publications were published during the last 5 years and only 7 before 2000. The absolute number of participants with HIV-related dementia ranged from 0 to 396, with a prevalence ranging from 0% to 80%.

**Table 3:** Overview of studies on HIV-related dementia and risk factors in sub-Saharan

**Author, year of publication**	**Country/setting**	**Design/study period**	**Population characteristics**	**Diagnostic criteria**	**Prevalence**	**Risk factors**	**Comments**
Belec [77], 1989	Central African republic, Hospital	Cross-sectional 1987	93 HIV + participants; age and sex not specified	Not reported	HAND: 3 cases (3.2%)	NA	No neuro-imaging or neuropathological studies
Howlet [78], 1989	Tanzania, hospital	Cross-sectional 1985-1988	200 (men 129) HIV + participants; mean age: 32 y	Decline of memory and other functions	Dementia complex: 54%	NA	
Turnbull [79], 1991	South Africa	Cross-sectional 1982-1983	27 haemophilic patients with HIV infection	Battery of neuropsychological tests: Rey complex figure, Babcock story, digit span, WAIS	HAND: 4 cases (14.8%)	NA	
Perriëns [80], 1992	Democratic republic of Congo Hospital	Cross sectional 2008	104 (men 48) HIV + participants; mean age: 34.3 y.; 92 (men 53) HIV- participants; mean age 44 y 9 (men 5) HIV + with HAND	WHO operational criteria/American Academy of neurology criteria	HIV Associated |Dementia Complex. 8.7%	NA	No neuro-imaging study
Maj [81], 1994	Kenya Hospital	Cross sectional 1990-1991	65 (men 49) HIV- participants; mean age: 30 y.; 66 (men 42) asymptomatic HIV + participants; mean age 30.7; 72 (men 48) symptomatic HIV + participants; mean age: 33.2 y	ICD-10/DSM-IV	Dementia HIV- 0 Asymptomatic HIV + 0 Symptomatic HIV + 6 (%)	NA	
	Democratic republic of Congo Hospital		85 ( men 48) HIV- participants; mean age: 33.9 y; 52 (men 33) asymptomatic HIV + participants; mean age 32.3 y.; 68 (men 35) symptomatic HIV + participants; mean age: 33.8 y	ICD-10/DSM-IV	Dementia HIV- 0 Asymptomatic HIV + 0 Symptomatic HIV + (5.9%)	NA	
Carson [82], 1998	Kenya	Cross sectional	78 (men 52) HIV + participants; mean age: 29.9 y.; 138 (men 114) HIV- participants; mean age 29.8 y.	Revised WAIS, Trails A and Trails B tests, Digit span, Delayed word and d recognition	NA	NA	No difference in neuropsychiatric test performance between HIV + and HIV-
	Hospital	1994					
Sebit [83], 1995	Kenya	Cross sectional	191 participants, 72 (men 48) symptomatic HIV + (mean age 33.2 y.), 66 (men 42) asymptomatic HIV + (mean age 30.7) and 65 (men 49) HIV- (mean age 30 y.)	WHO operational criteria/American Academy of neurology criteria	Mental disorders:	NA	No specific data for HIV associated neurocognitive disorders
	Hospital						
		1990-1991					
					Symptomatic HIV + 7.1%, Asymptomatic HIV + 4.5%, HIV -0		
	Democratic republic of Congo (DRC)/Hospital		190 participants, 68 (men 35) symptomatic HIV + (mean age 33.8 y.), 52 (men 33) asymptomatic HIV + (mean age 32.3) and 85 (men 48) HIV- (mean age: 33.9 y.)	WHO operational criteria/American Academy of neurology criteria	Mental disorders:	NA	No specific data for HIV associated neurocognitive disorders
					symptomatic HIV + 5.9%, asymptomatic HIV + 1.9%, HIV– 1.2%		
Sacktor [84], 2006	Uganda, Hospital	Prospective	23 (men 5) HIV + participants on	MSK HIV dementia scale IHDS	Baseline: Subclinical dementia 35%	NA	All participants had CD4 count ≤200 cells/mL and an IHDS ≤ 10 (suggestive of HAND)
		Cohort study					
		2004-2005					
			HAART (mean age 32.8 y.)				
			Re-assessment at 3 and 6 months.				
					Mild dementia 61%		
					At 3 (6) months: mild dementia 26% (4%)		
Sacktor [85], 2005	Uganda, Hospital	Cross-sectional 2003-2004	81 HIV+; mean age: 37 y.; 100 HIV- mean age: 31.4 y; 21 had HIV dementia	IHDS (cut off ≤10),	HIV dementia: 31%	NA	
				MSK HIV dementia scale			
Modi [86], 2007	South-Africa, Hospital	Cross-sectional	506 HIV + (men 203) on HAART; mean age/range: 37 years 193 had HIV associated dementia	American Academy of Neurology AIDS Task force	HIV dementia: 38%	NA	75% had CD4 below 100 cells/mm3
		2005					
Clifford [87], 2007	Ethiopia, Hospital	Case–control	73 (men 67%) HIV + participants (median age 39 y.);	IHDS	NA	NA	Quantitative neuropsychiatric tests - no difference between groups
		2004					
			87 (men 63%) HIV- participants (median age 38 y.)				
Odiase [88], 2007	Nigeria, Hospital	case–control	96 (men 48) symptomatic HIV + patients (mean age 33.6 y.),	FePsy computerized neuropsychological test battery	NA	NA	Severity of immune suppression predictive of cognitive decline
		2004					
			96 (men 48) asymptomatic HIV + (mean age 31.5 y.); 96 (men 48) HIV- (mean age 32.9 y.)				
Wong [89], 2007	Uganda, Hospital	Cross-sectional	78 (men 28) HIV + participants (mean age 37 y.); 24 (men 6) with dementia; 100 HIV – participants	MSK HIV dementia scale	HIV dementia. 31%	Age, low CD4 count associated HIV dementia	
		2003-2004					
Robertson [90], 2007	Uganda, Hospital	Cross-sectional	110 (men 34) HIV + participants (WHO Stage 2/3/4, n = 21/69/20); mean age 36.7 y.; 49 on HAART	MSK HIV dementia scale	NA	NA	Pattern of neuropsychological deficits similar to that in western countries.
		2003-2004					
			100 (men 60) HIV– controls (mean age 27.5 y.)				
Salawu [91], 2008	Nigeria, hospital	Cross-sectional	60 HIV + (men 24), asymptomatic, naïve of HAART; mean age 32 y)	CSID	56.7%	No correlation between CD4 count and performance on neuropsychological testing	
			60 HIV- (men 24); mean age: 30.1 y;				
			34 had HIV dementia				
Singh [92], 2008	South Africa, Hospital	Cross-sectional	20 HIV + (men 8) participants; median age 34 y	IHDS-criteria (cut-off ≤10)	HAND: 80%	NA	CD4 < 200 cells/mm3, older than 18 years and not be delirious.
		2007	16 had HAND				
Säll [93], 2009	South Africa, Hospital	Retrospective	38 HIV + admitted to the psychiatric ward with psychiatric symptoms; mmean age 32.4 y	DSM-IV	Dementia: 32%	NA	
		1987-1997					
			12 had dementia				
Ganasen [94], 2008	South Africa, Hospital	Cross-sectional	474 (men 123) HIV + patients (328 blacks and 135 coloured); mean age 34 y.	HIV dementia scale	HAND: 17.1% (IHDS) and 2.3% (MMSE)	NA	
				MMSE			
Njamnshi [95], 2008	Cameroon, Hospital	Case–control study 2006	204 (men 64) HIV + participants (mean age 37.2 y.); 204 (men 64) HIV- participants (mean age 37.1 y.)	IHDS-criteria (cut-off ≤10)	HAND:	NA	
					HIV+: 21.1%		
					HIV-: 2.5%		
Sacktor [96], 2009	Uganda, Hospital	Prospective cohort	102 (men 29) HIV + never treated patients (mean age 34.2 y.) started on Stavudine-based HAART	IHDS criteria	Base line: 40% had HIV dementia (33% mild, 7% moderate)	NA	
				MSK HIV dementia scale			
		2005-2007					
		Follow-up 6 months					
			25 (men 15) HIV- (mean age 30.3 y.)				
					At 3 months: 26%, 23% mild, 3% moderate		
					At 6 months: 16% (13% mild, 3% moderate		
Njamnshi [97], 2009	Cameroon, Hospital	Cross-sectional	185 (men 61) HIV + participants (mean age 37 y.); 41 with possible HAND (mean age 37y.)	IHDS-criteria	HAND: 22. 2%	Advanced clinical stage, low CD4 count, and low haemoglobin levels	
		2006					
Sacktor [98], 2009	Uganda,	Cross-sectional	60 HIV + never treated participants; 22 with dementia	IHDS criteria	Overall: 36.7%	HIV subtype D associated with increased risk of HIV dementia	All participants had CD4, count ≤200 cells/mL and an IHDS ≤ 10 (suggestive of HAND)
	Hospital						
		2005-2007					
				MSK HIV dementia scale			
Nakasujja [99], 2010	Uganda,	Prospective cohort	102 HIV + (men 28); mean age: 34.2 y; 70 with cognitive impairment at baseline	IHDS (cut-off ≤10)	Base line: 68.6%	NA	
	Hospital						
		2005-2007					
				neuropsychological tests and MSK HIV dementia scale	At 3 months: 36%		
					At 6 months: 30%		
Kinyanda [100], 2011	Uganda,	Cross-sectional	618 HIV + (men 169), 83% <45 y	IHDS (cut-off ≤ 10)	64%		
			396 had cognitive disorders				
	Hospital	2010					
Choi [101], 2011	Guinea Bissau,	Case–control	22 HIV-2 + (men 4)participants mean age for those with CD4 < 350 = 55.1 y, mean age for those with CD4 ≥ 350 = 50.3 y)	IHDS	HIV+: 22.7% (CD4 < 350 = 27%, CD4 ≥ 350 = 18%)	age (β = -0.11)	
	Hospital						
			45 HIV- controls (men 1); mean age51 · 9 y)	MSK HIV dementia scale			
					Control: 11%		
Birbeck [102], 2011	Zambia,,	Cross-sectional	496 HIV + (men 205) participants screened within 1 week of initiating ART; mean age 38.1 y)	I\HDS (cutt-off ≤ 10)	42.1% (IHDS)	NA	Low IHDS score was associated with poor adherence to HAART
	Hospital	2006-2007					
				MMSE (<=22)	34.4% (zMMSE)		
			IHDS administered to 440 participants.				
			185 had dementia				
Joska [103], 2010	South Africa, Hospital	Cross-sectional	536 (men 26.7%) HIV + participants (68% blacks, 28% coloured), mean age 34 y.	HDS (cutt-off ≤ 10)	HAND: 23.5%	Age, education, diagnosed duration, post-traumatic stress disorder	IDHS not yet available by the time of the study
Kanmogne [104], 2010	Cameroon	Case–control	43 (men 18) HIV- participants (mean age 33.3 y.); 44 (men 17) HIV + participants (mean age 34.9 y.); 22 with AIDs defining conditions, 34% on HAART	HIV Neurobehavioral Research Center International neuropsychological test battery	NA	NA	
	Hospital						
		2008-2009					
Lawler [105], 2010	Botswana,	Cross-sectional	120 (men 60) HIV + patients (mean age 37.5 y.); 97.5% on HAART;	IHDS-criteria (cut-off ≤9.5)	HAND: 38%	NA	
		2008	46 with HIV dementia				
	Hospital						
Patel [106], 2010	Malawi, Hospital	Cross sectional	179 (men 63) HIV + participants (mean age 36.7 y.); Stage III/IV 90%; 134 on HAART > 6 months;	IHDS-criteria (cut-off ≤10)	HAD	Female gender, low education	
		2007					
			25 (men 14) with HIV dementia				
					Overall: 14%		
					Men: 22.2%		
					Women: 9.5%		
Siddiqi [69], 2009	Zambia	Cross-sectional	443 (men 219) inpatients (median age 39 y., 67 HIV+); 368 (men 168) outpatients (median age 39 y., 58 HIV+); Overall 36 cases of dementia	Not specified	NA	HIV+: 10.4%	HIV + patient had a higher frequency of dementia and had dementia at younger age
	Hospital						
						HIV-: 3.3%	
Ekenze [21], 2010	Nigeria, Hospital	Cross-sectional	8440 admissions; 1249 (men 640) with neurological diseases (mean age 45 y.); 44 (men 18) with AIDS dementia complex	Not specified	AIDS dementia complex: 3.5% of all neurological admission	NA	
		2003-2007					
Holguin [107], 2011	Zambia, Hospital	Case–control	57 (men 30) HIV- participants (mean age 28 y.); 83 (men 32) HIV + (mean age 34 y.) including 54 naïve of HAART	IHDS (cut-off ≤ 10)	HAND = 22% among HIV + naïve of ARV	NA	
				Color Trails Test 1 and			
		2008		2, Grooved pegboard Test, and Time Gait Test			
Joska [108], 2011	South Africa, Hospital	Case–control	94 (men 36) HIV- participants (mean age 25.2 y); 96 (men 20) HIV + (mean age 29.8 y)	IHDS	NA	Education associated with IHDS total score	Validation study of the IHDS
		2008					
Obiabo [109], 2011	Nigeria,	Prospective Cohort study	69 (men 25) HIV + participants with CD4 < 350 (mean age 36.2 y.); 30 (men 11) HIV- (mean age 36.6 y.)	CSID and FePsy computerized neuropsychological test battery	NA	NA	HAART improved neuropsychological performances after 12 months of treatment
	Hospital						
Joska [110], 2011	South Africa Hospital	Cross-sectional	170 (men 44) HIV + participants (mean age 29.5 y.)never treated; 43 (men 14) with HIV-dementia; 72 (men 19 with MND	AAN revised criteria	Mild neurocognitive disorder: 42.4% HIV dementia: 25.4%	Education, and male gender independent predictors of HIV-dementia	
		2008-2009					
Robertson [111], 2011	Malawi,	Cross sectional	133 (men 39) never treated HIV + patients (median age 31 y.)	Not provided	MND: 8%		
					HAD: 0%		
	Hospital						
	South Africa,		167 (men 60) never treated HIV + patients (median age 34 y.)	Not provided	MND: 4%		
					HAD: 0%		
	Hospital						
	Zimbabwe, Hospital		80 (men 31) never treated HIV + patients (median age 36 y.)	Not provided	MND: 14%	NA	860 HIV + HAART naïve patients with CD4 count < 300 cells/mL and KI ≥70%
					HAD: 3%		
Robbins [112], 2011	South Africa,	Cross-sectional	65 (men 23) HIV + patients on HAART for ≥6 months (mean age 38.5 y)	IHDS and Xhosa-validated IHDS	HIV Associated dementia 80%	Low CD4 counts, alcohol dependency	
	Hospital	2009-2010					
Kwasa [113], 2012	Kenya,	Cross sectional	30 (men 17) HIV + patients (mean age 39 y.)	Neuropsychological test battery MMSE/IHDS (cut-off ≤10)	HAD 20%	NA	
	Hospital		6 (men 5)with HAD				
Spies [114], 2012	South-Africa,	Case–control	35 HIV + without childhood trauma; mean age: 31.5 y	Neuropsychological test battery	NA	NA	Significant HIV effects for the Hopkins Verbal Learning Test (HVLT) learning and delay trials and the Halstead Category Test (HCT)
	Hospital		48 HIV + with childhood trauma; mean age: 31.7 y				
			27 HIV- without childhood trauma; mean: 25y				
			20 HIV- with childhood trauma; mean age: 27 · 7 y				
			All participants were women.				
Hestad [115], 2012	Zambia, Hospital	Case–control	38 HIV + (men 16); mean age: 28.3 y 42 HIV- (men 18); mean age: 28.9 y	Neuropsychological tests	NA	NA	HIV + individuals performance lower than that of HIV- on verbal fluency, executive function, speed of information processing, verbal episodic memory and motor function
Berhe [116], 2012	Ethiopia,	Cross-sectional	347 HIV + (men 176) participants; mean age/range: 34.6 y admitted with neurological disorders	“cognitive and motor abnormalities, CT/MRI showing brain atrophy and other opportunistic infections ruled out”	HIV encephalopathy: 0.3%	NA	
	Hospital	Retrospective					
			10 had dementia				
		2002-2009					
Joska [117], 2012	South Africa,	Prospective	166 HIV + participants assessed at baseline, 108 reassessed at one year (82 received HAART)	Neuropsychological tests	NA	Lower level of education	Improvement on neuropsychological tests for all participants at one year.
				Average Global deficit score			
	Hospital						
Breuer [118], 2012	South Africa,	Cross-sectional	269 HIV + (men 97) participants on HAART for ≥6; months; 34% aged >40 y)	IHDS (cut-off ≤10.5)	HAND: 12%	NA	
	Hospital						
Hoare [119], 2012	South Africa	Cross-sectional	43 stage III HIV + (24 with at least one ϵ4 ApoE allele, men: 8, Age: 29 y and 19 without the ϵ4 ApoE allele, men: 2, Age: 28 y)	Neuropsychological test battery	NA	Performance on Hodgkin Verbal Learning Tool- Revised was poorer in the group with the ϵ4 genotype.	
						Participants with the ϵ4 genotype had more white matter injury on MRI.	
	Hospital						
Oshinaike [120], 2012	Nigeria	Case–control	208 HIV + (men 71), mean age: 36.8 y	IHDS (cut off ≤10)	HAND by MMSE: 2.9%	Lower CD4 count	
	Hospital	2007-2008					
			121 HIV – (men: 35), mean age:38.0 y	MMSE (cut off ≤26)			
				AAN revised criteria (any value below 2SD)			
					HAND by IHDS: 54.3%		
					HAND by AAN: 42.3%		
Royal [121], 2012	Nigeria, Hospital	Cross-sectional	60 (men 23) never treated HIV + participants (mean age 34 y);	IHDS (cut off ≤10)	28.8% HIV + individuals scored abnormally	Low CD4 count, WHO clinical stage of disease	
			56 (men 34) HIV- (mean age 29 · 4 y.); 32 had dementia				
					16.0% HIV- individuals scored abnormally		

3TC: Lamivudine; AIDS: Acquired Immunodeficiency Syndrome; CD4: cluster of differentiation 4; CSID: Community Screening Interview for Dementia; CT: computerized tomography; DSM-III-R: Diagnostic and Statistical Manual 3^rd^ edition revised; DSM-IV: Diagnostic and Statistical Manual 4^th^ edition; dT4: Didanosine; FePsy: The Ion Psyche Program; HAART: Highly Active Anti-Retroviral Treatment; HAD: HIV Associated Dementia; HAND: HIV Associated Neurocognitive Disorders; HDS: HIV Dementia Scale; HIV: Human Immunodeficiency Virus; ICD-III-R: International Classification of Disease; IHDS: International HIV Dementia Scale; MSK: Memorial Sloan Kettering; MMSE: Mini Mental State Examination; MND: Mild Neurocognitive Disorder; NA: Not available; NVP: Nevirapine; WHO: World Health Organization; y, years; ZDV: Zidovudine.

The diagnostic tools used to identify HIV-related dementia were variable, making comparison between studies less reliable. However, the International HIV Dementia Scale (IHDS) [89,95,97,105,107-110,112,113,120,121] and the Sloan Memorial Kettering scale [86,89,90,98] were frequently used. Studies that used the IHDS reported a prevalence ranging from 21.1 to 80%. The mean/median age of participants ranged from 31 to 40 years for those with HIV-related dementia, and men represented 25% to 56% of this group. In the nine studies that investigated etiological factors, the identified determinants of HIV-related dementia were: low level of CD4 count (four studies), low level of education, and advanced age (three studies), comorbid psychiatric conditions (two studies each), advance clinical stage (two studies), male sex, HIV-subtype and duration of disease (one study each). The most commonly reported risk factors of HIV associated dementia were the level of CD4 count [89,97,112,120,121] and the clinical stage of disease [97,121].

### Amyotrophic lateral sclerosis and cerebellar degeneration

Fifteen studies (12 retrospective, 2 cross-sectional and 1 case-series) (Table 4) including 13 hospital and two community-based studies on amyotrophic lateral sclerosis (ALS) have been conducted in 9 SSA countries including Nigeria (four studies), Senegal (three studies), Ethiopia (2 studies), Zimbabwe, Kenya, South Africa, Sudan, Cameroon and Ivory coast (one study each). The number of participants with ALS ranged from two to 73. Two community-based studies provided a prevalence of 15/100,000 and 5/100,000 respectively in Nigeria [19] and in Ethiopia [122]. Five hospital-based studies provided prevalence figures: between 0.2 and 8.0/1000 of all neurologic consultation/admission [16,21,122-126]. The method of ascertainment of ALS was variable across studies, but electromyography was done in four of the fifteen studies included [125-129]. The proportion of men among those with ALS was 57.6 to 100%. The age of those with ALS ranged from 12 to 84 years. When provided, the age at the clinical onset of ALS ranged from 12 to 71 years and the time to diagnosis from 3 months to more than 15 years. In general, risk factors for ALS were not investigated across studies.

**Table 4:** Overview of studies on amyotrophic lateral sclerosis risk factors in sub-Sahara Africa

**Author, year of publication**	**Country/setting**	**Design/year**	**Population characteristics**	**Diagnostic criteria/tools**	**Prevalence**	**Risk factors**	**Comments**
Wall [130],1972	Zimbabwe	Retrospective	13 (men 10) consecutive patients; age 24–55 y.	Clinical (no ENMG)	NA	NA	6 participants had sensory changes
	Hospital-based	1967-1971					
Osuntokun [126], 1974	Nigeria	Retrospective	92 patients with MND ALS 73; PMA 10, SMA 9	ENMG/Muscle biopsy/	21/100,000	NA	Mean age at onset: 39 y
							Mean duration of disease exceeded 15 y in 8% of participants
	Hospital-based	1958 -1973					
							4 patients with ALS had poliomyelitis in childhood.
Osuntokun [19], 1987	Nigeria	Cross-sectional	18954 participants (men 9282); 58% <20 y and 11% > 50 y	Screening questionnaire developed by the authors	MND: 15/100,000	NA	
	Community-based	1985					
Cosnett [125], 1989	South Africa Hospital-based	Retrospective Cases collected during 9.5 y.	59 blacks (mean age 47.4 y.); 16 whites and 2 coloured (mean age 54 y.) 9 Indians (mean age 54 y)	Clinical and ENMG in 45%	Blacks/white & coloured/Indians (per 100,000) 0.88/2 · 7/1.4	NA	Mean age of onset: 47 y (blacks) and 54 y (in whites and Indians)
							29% of participants not followed up.
Ekenze [21], 2010	Nigeria	Retrospective	8440 admissions; 1249 (men 640) with neurological diseases, mean age 45 y.; 10 (men 4) with ALS	Not specified	800/100,000	NA	
	Hospital-based	2003-2007					
Abdulla [127], 1997	Sudan	Retrospective:	28 (men 17) patients with MND; 19 (men 14) with ALS	Clinical and ENMG	NA	Family history of MND in 14%	Mean age of onset: 40 y
	Hospital-based	1993-1995					
Kengne [16], 2006	Cameroon	Retrospective	4041 neurologic consultations; 145 with neurodegenerative diseases 10 (men 8) with ALS; mean age 50.9 y.	Not provided	12% of all neurodegeneration 250/100,000 of all neurologic consultation		4 selected degenerative brain diseases: Dementia, PD, ALS and chorea
	Hospital-based	1993-2001					
Imam [131], 2004	Nigeria	Retrospective	16 (men 15) participants; age 16-60 y.	El Escorial diagnostic criteria for ALS, no ENMG	NA	NA	
	Hospital-based	1980-99					
Adam [129], 1992	Kenya	Retrospective	47(men 35) participants with MND;	Clinical (ENMG in 1/3 of participants)	NA	NA	Duration of disease: 5 m to 4 y.
	Hospital-based	1978-88	Age 13-80 y				
			18 had ALS				
Tekle-Haimanot [122], 1990	Ethiopia	Cross-sectional	60820 participants (men 29412), 59% aged < 20 y	Screening questionnaire and neurological exam	5/100,000	NA	A population survey of neurological diseases
	Community-based	1986-88	3 (2 men) had MND				
Harries [132], 1955	Ethiopia	Case series	2(all males) participants	Clinical (no ENMG)	NA	NA	
			Age 26 and 30 y				
	Hospital-based	1954					
Jacquin-cotton [123], 1970	Senegal	Retrospective	6100 participants with neurological disorders	Clinical (No ENMG)	290/100,000		A study of patients with paraplegia in a neurological unit
	Hospital-based	1960-1969	18 (16 men) participants with ALS, age 25-70 y				
Piquemal [124], 1982	Ivory coast	Retrospective	4000 participants with neurological disorders	Clinical (no ENMG)	750/100,000	NA	Duration of disease: 3 m to 5 y.
	Hospital-based	1971-80	30 (men 22) participants had ALS, 50% aged <40 y				
Collomb [133], 1968	Senegal	Retrospective	18 (17 men) participants with ALS, age 25-70 y	Clinical (no ENMG)	NA	NA	Duration of disease: 4 m to 13 y
	Hospital-based	1960-68					
Sene [128], 2004	Senegal Hospital-	Retrospective	33 (19 men) participants with ALS;	El Escorial			Definite ALS: 57%,
							Probable: 30%, Possible ALS: 9%
							Suspect ALS: 3% age at onset 14–67 y.
				(ENMG in half of the patients)			
	based	1999-2000					Duration of disease: 6 m to 5 y.

ALS: amyotrophic lateral sclerosis; ENMG: Electroneuromyography; MND: Motor Neuron Disease; NA: Not available; PMA: Progressive muscular atrophy; SMA: Spinal Muscular Atrophy; y: years; m: months.

One retrospective study in Nigeria reported on two cases (a 32 year old male and a 42 year old female) of cerebellar degeneration among 2 · 1 million admissions over a period of 25 year [14]. One study in Rwanda reported on a family of 33 members, with 15 (including eight men, age at onset 12–49 years) having type 2 spino-cerebellar ataxia [134]. A study in Mauritania reported on 12 cases of cerebellar degeneration-based on clinical criteria, including 9 familial cases (including 7 men, aged 3 to 29 years) and 3 apparently sporadic cases (all men, aged 8 to 50 years) [135]. Another clinic-based study of paraplegia in Senegal reported on 7 cases of spino-cerebellar degeneration among 6100 neurological admissions [123].

### Huntington disease

Nineteen studies (four community-based studies and 15 hospital-based) investigated Huntington disease; including 8 cross-sectional studies (including reviews of medical records), 10 case series (two to 13 patients), and one case report (Table 5). The studies were conducted in nine countries: South Africa (nine studies), Zimbabwe and Tanzania (two studies each), Nigeria, Mauritius Island, Senegal, Sudan, Togo and Burkina Faso (one study each). The diagnostic of Huntington disease was mostly clinical, based on a constellation of probing clinical elements; however genetic testing was carried out in five studies [136-140]. The absolute number of participants with Huntington disease ranged from one to 481. Only one community-based study provided a prevalence estimate of 3.5/100,000 in South-Africa [141]. The hospital-based prevalence of Huntington disease when reported ranged from 0.2/100,000 to 46.0/100,000 [138,142-146]. No study reported data on the incidence of Huntington disease. Among those with the disease, males represented 42 to 100%, and age varied from <9 years to 80 years. When provided, the age at the clinical onset of the disease ranged from less than one year to 58 years. In general, antecedent risk factors for Huntington disease were not investigated across studies except for a positive family history reported in 58.3 to 100% of cases.

**Table 5:** Overview of studies on Huntington disease and risk factors in sub-Sahara African countries

**Author, year of publication**	**Country**	**Setting**	**Design/year of the study**	**Population characteristics**	**Diagnostic tool/criteria**	**Prevalence**
Hayden [141], 1977	South Africa	Community	Cross-sectional	26 cases (men 11); age 12–68 y.	Clinical	3.5/100,000
Samuels [147], 1978	Zimbabwe	Community	Case series	1 family of HD	Clinical	NA
				4 cases (men 2) age 14–26 y.		
Glass [148], 1979	South Africa	Community	Case series	2 cases of HD (men 1) age 42-52	Clinical	NA
Hayden [142], 1980	South Africa	Community/hospital	Cross-sectional,	481 cases (m en 241) of whom 153 (m en 69) alive by the time of the study	Clinical	Overall: 0.65/100,000, Whites: 2.22/100,000, Mixed ancestry: 2.17/100,000, Blacks: 0 · 01/100,000
Scrimgeour [149], 1981	Tanzania	Community	Case series	11 cases, aged 25–80 y.	Clinical	NA
Hayden [143], 1981	Mauritius	Hospital	Cross-sectional	2166 persons, 6 cases of HD (men 3)	Not provided	46/100,000
Hayden [144], 1981	South Africa	Hospital	Cross-sectional/NR	17 children (onset before 20 y.) identified during a national survey among of 219 patients	Not provided	Overall: 0.6/100,000
						Whites: 0.37/100,000
						Mixed ancestry: 0.89/100,000
						Blacks: No case
Hayden [150], 1982	South Africa	Community/hospital	Cross-sectional	157 (men 71) individuals investigated and 328 (women 156, only 3 negro-Africans) deceased individuals with probably HD	Not specified	Combined white and black heterozygote frequency = 6 · 7 x 100,000
Scrimgeour [151], 1982	Tanzania	Hospital	Case series (National registry)	7 patients with chorea (1 aged 80 y.) and 50 potential patients with chorea in 23 families	Not specified	NA
				Mean age at onset: 36 y.		
Aiyesimoju [145], 1984	Nigeria	Hospital	Cross sectional 1957-1982	2.1 million patients admitted to the hospital.	Not specified	HD: 0.2/100,000
				4 cases (men 3) of HD aged 24–50 y at diagnosis.		
Stephany [146], 1984	Senegal	Hospital	Cross sectional	12370 patients seen in a neurologic clinic; 3 (men 2) with HD; age 31–64 y.	Family history	24.2/100,000
					All patients had movement disorders and neuropsychiatric features	
			1960-1980			
Joubert [136], 1988	South Africa	Community/hospital	Cross-sectional 1983-1986	8 cases in hospital setting (n = 6. all men) and at home (n = 2);	Clinical/genetic testing/screening for Wilson disease	NA
				Age at onset: 8–47 y.		
				Age at diagnosis: 13–50 y.		
Scrimgeour [152], 1992	Zimbabwe	Hospital	Case series1991	11 cases in a 4 generation of a single family; 2 probable cases	Clinical	0.5/100,000
Scrimgeour [153], 1995	Sudan	Hospital	Case-report	1 case of HD: A	Clinical/MRI	NA
				40 year old black Sudanese man		
Grunitzky [154], 1995	Togo	Hospital	Case series	A family including 8 patients with HD and 67 at risk across 6 generations; mean age at onset: 33 y.	Not specified	NA
Silber [137], 1998	South Africa	Community	Case series	5 families of HD including a total of 7 genetically confirmed cases of HD and 10 clinically suspect cases of HD	Clinical/genetic testing	NA
Kabore [138], 2000	Burkina-Faso	Hospital	Case series	4 cases of HD; age at diagnosis 33–43 y.	Clinical/genetic testing	0.04/100,000
Bardien [139], 2007	South Africa	Hospital	Case series 2001-2005	A family with HD like 2	Clinical/genetic testing	1
				Total 39 family members		
				13 had the disease		
Magazi [140], 2008	South Africa	Hospital	Case series	12 cases (men 6); age 25–52 y.	Clinical/genetic testing	NA

HD; Huntington disease; MRI: magnetic resonance imaging; NA: not applicable; NINCDS-ADRDA, National Institute of Neurological and Communicative Disorders and Stroke and the Alzheimer’s Disease and Related Disorders Association; y: year.

## Discussion

This review represents an unprecedented effort to summarize epidemiological data on neurodegenerative diseases in SSA. However, this being a large diverse multicultural and multiethnic region, it is difficult to reliably quantify and compare the burden of neurodegenerative disorders across countries. Although mostly based on prevalent cases and on retrospective data, from studies that have essentially included urban populations, findings summarized in the current review are very informative.

The most widely investigated and prevalent neurodegenerative condition appeared to be dementia with most cases being of Alzheimer disease type. Major risk factors of AD include an advanced age (higher after the age of 60), female sex, a low schooling (less than 6 year of education), family background and rural residence. Unlike North America, Australia, Europe, and Japan where several population-based studies have been conducted on dementia, good quality epidemiological studies (prospective, population-based, using standardized criteria) are scanty in SSA, with methodological issues hampering any meaningful comparison with other regions of the world. The reported prevalence in one collaborative good quality study in Nigeria about 20 years ago among those aged >60 years was 2.3%. This was lower than the reported prevalence in developing countries, but within the range of reports from developing countries in Asia and Latin America where reported prevalence range from 1.9 to 3.8% [155]. The anticipated ageing of the population (which is the main driver of dementia figures) in Africa may translate in a higher prevalence and absolute number of people living with dementia as observed in other developing regions. However, caution is needed when interpreting findings from studies conducted in different settings by different investigators. Our overview tends to suggest that the projected increase in the prevalence of dementia in SSA is likely, based on the comparison of findings from three recent studies with those from the study above conducted in Nigeria 20 years ago [55-57]. Furthermore, with the large scale implementation of antiretroviral therapy and related improved survival, it is expected that the number of patients with the diagnosis of HIV-related neurocognitive impairment may increase as suggested by the increasing number of related-publications. Such trends will need to be confirmed by large scale prospective observational studies which will also assess the putative accelerating effect of HIV-related neurocognitive impairment on other types of prevalent dementia and neurodegeneration.

For Parkinsonism, the wide prevalence range observed both in population and hospital-based studies might also be a consequence of differences in methodologies for case ascertainment, diagnostic criteria, or age distributions of the study populations. These heterogeneities in PD prevalence are not unique to SSA as these have also been observed in Europe where prevalence of PD ranged from 66 to 12,500/100,000 [156]. There have been provisional set of minimal scientific criteria for conducting epidemiological studies on PD which, when adopted at a large scale will improve comparison within SSA and between SSA and other regions of the world [156]. Prevalence rates reported in population-based studies in the continent are limited to two studies and cases were ascertained through screening and neurological exam in one study, thus making any comparison with other region difficult. In ALS and Huntington disease, the picture is less clear as the majority of studies were hospital-based, retrospective in nature, with a final diagnosis not always based on pathology or genetics and the risk factors not properly assessed; thus making comparisons and inferences inaccurate. For these two conditions therefore, important gaps remain to be filled, without which the issues of prevention and control will not be efficiently addressed in the African context.

The comparatively higher number of population-based investigations of dementia relative to other neurodegenerative conditions in SSA, may at least in part be explained by the availability of standardized and widely accepted screening and diagnostic tools/criteria which facilitate epidemiological studies of dementia [157] as compared with other conditions where existing tools have not always been validated in different settings and therefore remain unpopular [158,159], or which, by the virtue of their low prevalence makes any assessment in the general population difficult and very expensive. There are context-specific challenges to obtaining key epidemiological data on neurodegenerative conditions in SSA including the low level of patient education, the need to accurately translate available screening and diagnostic tools to local languages, limited number of scientists and clinicians in neurosciences, and competing health interest in the setting of limited financial resources [5,16].

### Needs in terms of epidemiological data

In order to improve the knowledge base of each of the neurodegenerative conditions addressed in this review, two main types of epidemiological studies appear necessary and feasible in SSA. A population-based prevalence and incidence study including both urban and rural populations, in order to capture the real variability in socio-economic status and possibility in other factors that may exist in the population. Such a study may serve a dual purpose, providing information on disease rate and identification of key risk factors, as it would permit to establish the sequence of events. Given that such an undertaking could be planned beforehand, it offers the possibility of addressing multiple questions and/or diseases at a reduced cost. Inclusion of a large enough but manageable number of participants would be necessary to ensure adequate precision around the estimates generated. As many patients with possible neurodegenerative conditions would be tempted to consult traditional healers rather than accessing health facilities in SSA, special efforts would be required to ensure that these people are captured by such a study. Also, ascertaining cases of neurodegenerative conditions in a population-based sample may be costly and logistically challenging, particularly with regard to the asymptomatic or mildly symptomatic nature of early stages of some of the diseases, and the lack of validated instruments and appropriate expertise.

A second type of epidemiological study is a multicenter, hospital-based, registry investigation. The latter has several advantages over a single large-scale cohort study. Large numbers of cases could potentially be collected over a relatively short period of time, with the possibility of comparing resources and outcomes within and across countries. However, the major limitations of this approach include the costs associated with the effort and infrastructure for coordination and communication between centers, as well as data capture and ongoing monitoring and quality control. In addition, there are biases inherent to any such hospital-based study, especially given that in SSA there is major access and cost barriers to care, with a sizeable proportion of patients with neurodegenerative conditions who are never seen by health care providers thus limiting the scope of registries. The degree of such selection bias is likely to vary considerably across centers, affecting both case mix and outcomes. The approach would therefore not provide a study population fully representative of incident cases and the natural history of disease and its management.

For both types of studies, the definition of the pool of people ‘at-risk’ population could be challenging in the SSA context, given the lack of formal census of the population in many countries; thus making reliable estimation of the effect of individual risk factors difficult. Other methodological issues relate to the assessment of the outcome in a reliable fashion in the African context as discussed above. Hence, a combination of the aforementioned study approaches would probably overcome some of their respective limitations and improve the quality of estimates generated.

The challenges to performing high quality incidence and prevalence studies of neurodegenerative diseases are well known [159]. Cases of most neurodegenerative conditions are difficult to define and ascertain reliably in population-based sample, and there are problems in relating events and the effects of different exposures to defined ‘at-risk’ populations. With the ageing of the population in SSA, the importance of HIV/AIDS, as well as the surge in risk factors such as hypertension and diabetes that have been linked to dementia [157,160,161] and possibly to Parkinson diseases [162,163], the importance of neurodegenerative disorders would considerably increase over time. Indeed, by 2025, the numbers of people aged 60 years and over will more than double in many countries [164]. With this rapid demographic and nutritional transition, neurodegenerative conditions would become an important public health problem in SSA. Critical investments are therefore necessary to improve surveillance and program-relevant research to provide an evidence base for policy development and effective control and prevention of neurodegenerative diseases. Precise identification of risk factors other than ageing would allow proper prevention effort spanning from primordial to secondary and event tertiary prevention, given that most of those conditions are associated with higher levels of disability and increased risk of death. Community-based risk factor control, combined with high risk approaches and realignment of health systems to incorporate the chronic management of neurodegenerative diseases are needed.

### Strengths and limitations of the review

Our review is the first of its kind on neurodegenerative conditions in SSA. It is more up-to-date and broader than previous attempts to summarize evidence on single diseases in this setting [4-8]. By systematically assessing all published articles on these conditions, we aimed to draw the attention on the importance of the conditions in the region, and identify the research priorities. A limitation of this review is inherent to the limitations of the individual studies included. We relied on clinic-based studies where necessary in this systematic review; but such studies have limitations, particularly with regard to the generalization of their results data. However, we have tried to convey a clear understanding of the current burden and risk factors of each condition by examining all published papers across a broad range of clinical, biology, public health, and psychosocial literature, incorporating various types of evidence. By the nature of the disease, the age range for participants in studies on ALS and HIV-related neurocognitive impairment extended to the pediatric age for some studies. It is of note that large number of studies are realized in hospital in Africa, often published in local journals or reported in thesis. It the absence of straightforward strategies for capturing this sort of evidence in a systematic way, we did not account for them, which may have lowered the number of results found in some countries. Finally, the many sources of heterogeneity precluded any meaningful assessed of the quality of the included studies.

## Conclusion

This review summarizes the body of literature on neurodegenerative disorders in SSA, which is large with regard to Dementia and HIV-related neurocognitive disorders but limited for other neurodegenerative disorders. In addition, it emphasizes some of the challenges in conducting good quality, population-based studies on the continent including the lack of standardized criteria for some neurodegenerative disorders, with most studies limited to few regions/countries on the continent. High-quality prospective cohort studies, which would use internationally- validated criteria, wide catchment areas in several geographic regions, and adjust for the projected ageing of the continent population, by compensating for the imprecise nature of the available data, will help map the epidemiology of neurodegenerative diseases in SSA and improve comparisons with the rest of the world.

## Competing interest

The authors declare that they have no competing interests.

## Authors’ contribution

All authors equally contributed. All authors read and approved the final manuscript.

## Pre-publication history

The pre-publication history for this paper can be accessed here:

http://www.biomedcentral.com/1471-2458/14/653/prepub

## Supplementary Material

Additional file 1 Search terms and strategies. Click here for file
