# Supplementary material for: Epidemiology of neurodegenerative diseases in sub-Saharan Africa: a systematic review
Source: BMC Public Health. 2014 Jun 26;14:653. doi: 10.1186/1471-2458-14-653 (PMC4094534; doi:10.1186/1471-2458-14-653)
Supplement: Additional file 1 — Search terms and strategies. [file 1471-2458-14-653-S1.doc]

**Additional file 1: Search terms and strategies**

| **Medline via PubMed (from inception to until February 2013)** |
| --- |
| **#**1 “Africa South of the Sahara" OR "sub-Saharan Africa”  #2 ( Angola OR Benin OR Botswana OR "Burkina Faso" OR "Upper Volta" OR Burundi OR Urundi OR Cameroon OR Cameroons OR "Cape Verde" OR "Central African Republic" OR Chad OR Comoros OR "Comoro Islands" OR Comores OR Mayotte OR Congo OR Zaire OR "Cote d'Ivoire" OR "Ivory Coast" )  #3 ( "Democratic Republic of the Congo" OR Djibouti OR "French Somaliland" OR Eritrea OR Ethiopia OR Gabon OR "Gabonese Republic" OR Gambia OR Ghana OR "Gold Coast" OR Guinea OR Kenya OR Lesotho OR Basutoland OR Liberia )  #4 ( Madagascar OR "Malagasy Republic" OR Malawi OR Nyasaland OR Mali OR Mauritania OR Mauritius OR Mozambique OR Namibia OR Niger OR Nigeria)  #5 (Rwanda OR "Sao Tome" OR Seychelles OR Senegal OR "Sierra Leone" OR Somalia OR "South Africa" OR Sudan OR Swaziland OR Tanzania OR Togo OR "Togolese Republic" OR Uganda OR Zambia OR Zimbabwe OR Rhodesia)  #6 (neurodegeneration OR “neurodegenerative diseases” OR “neurodegenerative disease”)  # 7 (dementia OR “Alzheimer’s disease” OR “fronto-temporal dementia” OR “Lewy body dementia” OR “cortico-basal degeneration” OR “multi system atrophy” OR “vascular dementia”)  # 8 (“Parkinson’s disease” OR parkinsonism OR “Parkinson’s syndrome”  #9 (“amyotrophic lateral sclerosis” OR “ALS” OR “motor neuron disease”)  # 10 (“Huntington disease” OR “Huntington chorea” OR “cerebellar degeneration”)  # 11 (“Complex, AIDS Dementia” OR “Dementia Complex, AIDS” OR “AIDS-Related Dementia Complex” OR “AIDS Related Dementia Complex” OR “HIV-Associated Cognitive Motor Complex” OR “HIV Associated Cognitive Motor Complex” OR “Dementia Complex, AIDS-Related” OR “ Complex, AIDS-Related Dementia” OR “Dementia Complex, AIDS Related” OR “HIV Dementia “ OR “Dementia, HIV” OR “Dementias, HIV” OR “HIV Dementias” OR “Acquired-Immune Deficiency Syndrome Dementia Complex” OR “Dementia Complex, Acquired Immune Deficiency Syndrome” OR “HIV-1-Associated Cognitive Motor Complex” OR “HIV 1 Associated Cognitive Motor Complex” OR “HIV-1 Cognitive and Motor Complex” OR “HIV 1 Cognitive and Motor Complex” OR “HIV Encephalopathy” OR “Encephalopathies, HIV” OR “HIV Encephalopathies” OR “Encephalopathy, HIV” OR “AIDS Encephalopathy” OR “AIDS Encephalopathies” OR “ Encephalopathies, AIDS” OR “Encephalopathy, AIDS” OR “HIV associated neurocognitive disorder” OR “AIDS dementia complex” OR “HIV associated dementia” OR “HIV-associated dementia” OR “Neuropsychiatric HIV”  **#**12 (#1 OR #2 OR #3 OR #4 OR #5)  #13 (#6 OR #7 OR #8 OR #9 OR #10 OR #11)  #14 (#12 AND #13) |
| **Banque des Données en Santé Publique (BDSP, from inception to until February 2013)** |
| #1 “Afrique au sud du Sahara” OU “Afrique sub-Saharienne”  #2 (Angola OU Bénin OU Botswana OU “Burkina Faso” OU “Haute Volta” OU Burundi OU Urundi OU Cameroun OU “Cap Vert” OU “République Centre Africaine” OU Tchad OU Comores OU “Iles Comores” OU Mayotte OU Congo OU Zaïre OU “Côte d'Ivoire”)  #3 (“République Démocratique du Congo” OU Djibouti OU “Somalie Française” OU Érythrée OU Éthiopie OU Gabon OU “République Gabonaise” OU Gambie OU Ghana OU “Gold Coast” OU Guinée OU Kenya OU Lesotho OU Basutoland OU Liberia)  #4 (Madagascar OU “République Malgache” OU Malawi OU Nyasaland OU Mali OU Mauritanie OU “Iles Maurice” OU Mozambique OU Namibie OU Niger OU Nigeria)  #5 (Rwanda OU “Sao Tome” OU Seychelles OU Senegal OU “Sierra Leone” OU Somalie OU “Afrique du Sud” OU Soudan OU Swaziland OU Tanzanie OU Togo OU “République Togolaise” OU Ouganda OU Zambie OU Zimbabwe OU Rhodésie)  #6 (neurodégénérescence OU “Maladie neurodégénérative” OU “Maladies neurodégénératives”)  #7 (démence OU démences OU “Maladie d'Alzheimer” OU “Démence fronto-temporale” OU “ Démence à Corps de Lewy” OU “Dégénérescence cortico-basale” OU “Atrophie mulstisystématisée” OU “Atrophie mulsti-systématisée” OU “Atrophie mulstisystémique” OU “Atrophie mulsti-systémique” OU “démence vasculaire”)  #8 (“Maladie de Parkinson” OU parkinsonisme OU “Syndrome Parkinsonien”)  #9 (“Sclérose larérale amyotrophique” OU “SLA” OU “Maladie du motoneurone”)  #10 (“Maladie de Huntington” OU “Chorée de Huntington” OU “Dégénérescence cérébelleuse”)  #11 (“Démence, SIDA” OU “Démence liée au VIH” OU “Troubles cognitifs liés au VIH” OU “Troubles psychomoteurs liés au VIH” OU “Démence du VIH” OU “Démence, VIH” OU “Démences, VIH” OU “Démences du VIH” OU “Démence du Syndrome de l'Immunodéficience Acquise” OU “Troubles cognitifs liés au VIH-1” OU “Troubles psychomoteurs liés au VIH-1” OU “Troubles cognitifs et moteurs liés au VIH-1” OU “Encéphalopathie liée au VIH” OU “Encéphalopathies, VIH” OU “Encéphalopathies du VIH” OU “Encéphalopathie, VIH” OU “Encéphalopathie du SIDA” OU “Encéphalopathies du SIDA” OU “ Encéphalopathies, SIDA” OU “Encéphalopathie, SIDA” OU “Troubles neurocognitifs associés au VIH” OU “Démence associée au VIH” OU “Manifestations Neuropsychiatriques du VIH”)  **#**12 (#1 OU #2 OU #3 OU #4 OU #5)  #13 (#6 OU #7 OU #8 OU #9 OU #10 OU #11)  #14 (#12 ET #13) |
